# A dosimetric study on the use of 3D‐printed customized boluses in photon therapy: A hydrogel and silica gel study

**DOI:** 10.1002/acm2.12489

**Published:** 2018-11-07

**Authors:** Yuehong Kong, Tengfei Yan, Yanze Sun, Jianjun Qian, Gang Zhou, Shang Cai, Ye Tian

**Affiliations:** ^1^ Department of Radiotherapy and Oncology The Second Affiliated Hospital of Soochow University Suzhou China; ^2^ Institute of Radiotherapy and Oncology Soochow University Suzhou China

**Keywords:** 3D printing, tissue compensator, silica gel, hydrogel, radiotherapy

## Abstract

**Purpose:**

The aim of the study was to compare the dose differences between two kinds of materials (silica gel and hydrogel) used to prepare boluses based on three‐dimensional (3D) printing technologies and commercial bolus in head phantoms simulating nose, ear, and parotid gland radiotherapy.

**Methods and materials:**

We used 3D printing technology to make silica gel and hydrogel boluses. To evaluate the clinical feasibility, intensity modulated radiation therapy (IMRT) plans were created for head phantoms that were bolus‐free or had a commercial bolus, a silica gel bolus, or a hydrogel bolus. Dosimetry differences were compared in simulating nose, ear, and parotid gland radiotherapy separately.

**Results:**

The air gaps were smaller in the silica gel and hydrogel bolus than the commercial one. In nose plans, it was shown that the *V*_95%_ (relative volume that is covered by at least 95% of the prescription dose) of the silica gel (99.86%) and hydrogel (99.95%) bolus were better than the commercial one (98.39%) and bolus‐free (87.52%). Similarly, the homogeneity index (HI) and conformity index (CI) of the silica gel (0.06; 0.79) and hydrogel (0.058; 0.80) bolus were better than the commercial one (0.094; 0.72) and bolus‐free (0.59; 0.53). The parameters of results (HI, CI, *V*_95%_) were also better in 3D printing boluses than in the commercial bolus or without bolus in ear and parotid plans.

**Conclusions:**

Silica gel and hydrogel boluses were not only good for fit and a high level of comfort and repeatability, but also had better parameters in IMRT plans. They could replace the commercial bolus for clinical use.

## 1 INTRODUCTION

In the radiotherapy of skin carcinomas, parotid gland carcinomas, or chest wall after mastectomy in breast carcinomas, there is a deficiency in the dose of the skin or superficial tissue due to the dose build‐up effect of the high energy X (γ) ray. The bolus can effectively improve the skin dose from 10% to 40% to nearly 100% in 6 MV X‐ray radiotherapy.^1^ The commercially available flat‐form boluses (hereafter, “commercial boluses”) are commonly used in the clinic, but they are difficult to make full contact with irregularly shaped patient skin. This lack of contact can result in the presence of air cavities between the bolus and the patient skin, which can compromise the accuracy of the surface dose.^1^, ^2^, ^3^ Conventional boluses include wet gauze, Vaseline, Truwax, Polyflex colloid, Jeltrate Plus, thermoplastic film, among others.^4^ They can be made into a suitable shape as needed to partially solve the air gap problem, but the existence of defects such as rough process, poor tissue uniformity, poor repeatability, and being easily moved, leads to a great deal of uncertainty in the actual radiation dose of superficial lesions, thus affecting the treatment efficacy. However, the use of three‐dimensional (3D) printing technology could help to create a patient specific bolus which facilitates correspondence with the patient skin, yielding agreement between the planned and delivered doses, but few studies have evaluated the silica gel and hydrogel bolus based on 3D‐printed technology in photon radiation therapy to our knowledge.

In this study, the bolus shells were fabricated by 3D printing technology and silica gel and hydrogel were selected as filling materials. The detailed physical characteristics and clinical feasibility of silica gel and hydrogel bolus were compared in simulating nose, ear, and parotid gland radiotherapy separately.

## 2 MATERIALS AND METHODS

### 2.A Bolus materials

The commercial bolus was Bolx‐I (CIVCO Medical Solution, Orange City, FL, USA) with a density of 1.03 g/cm^3^ and a main component of polymer gel. The boluses based on 3D printing technology were composed of silica gel or hydrogel. Silica gel was based on α, ω‐dihydroxypolysiloxane, ethyl polysilicate as the cross‐linking agent, and chloroplatinic acid as catalyst and synthesized at room temperature. The monomer of hydrogel was methacrylic acid and was initiated by ultrasonic polymerization.

### 2.B Physical property verification

Two kinds of boluses were placed on the standard solid water phantom computed tomography (CT) scanning was performed and then exported to a treatment planning system (Pinnacle version 9.8). Subsequently, the mean density of the bolus was measured randomly by 10 points. To evaluate the physical characteristics of the boluses, percentage depth dose (PDD) curves of two kinds of different materials were measured with a standard solid water phantom. The photon beam energy was set to 6 MV (Elekta Synergy) and the prescribed dose to the reference point was set to 100 MU with a 10 × 10 cm field, source to surface distance of 100 cm. The dose distributions and PDD curves were calculated using the treatment planning system (TPS) for each plan obtained from the physical evaluation with the water‐equivalent phantom. The calculated doses, *D*_0.5cm_, *D*_1cm_, *D*_1.5cm_, *D*_1.8cm_, *D*_2.0cm_, *D*_2.5cm_, *D*_3.5cm,_ and *D*_5.5cm_ were compared for each case.

### 2.C Bolus fabrication

**Figure 1:**
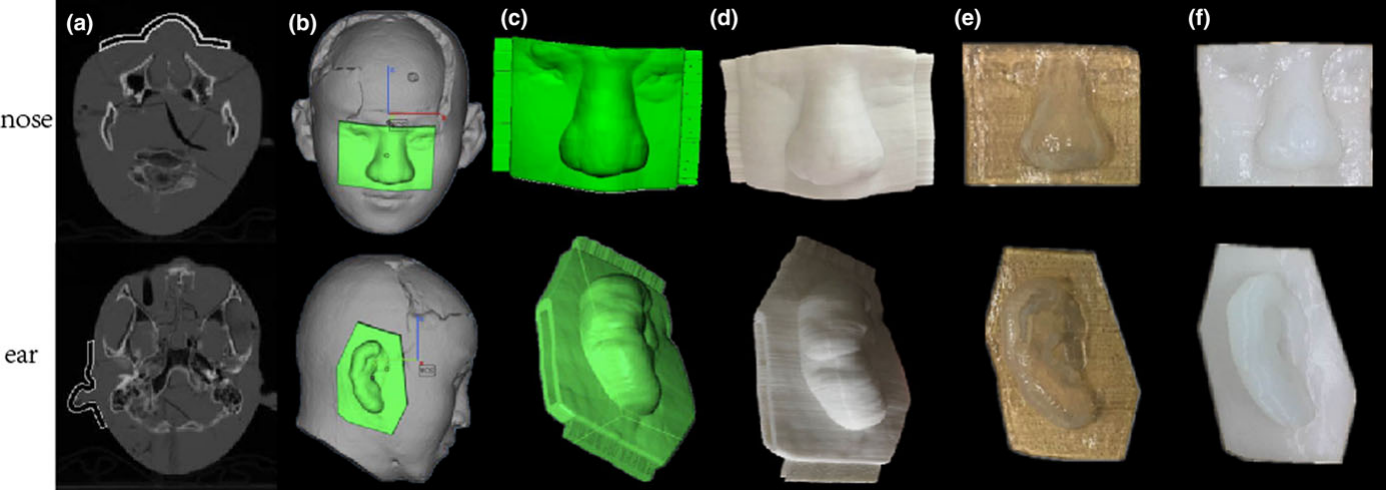
The procedure of making hydrogel and silica gel boluses based on 3D printing technology. (a) Virtual bolus structure created in the TPS, (b) the reconstructed and optimized bolus, (c) the designed bolus shell, (d) the bolus shell fabricated by 3D printing, (e) hydrogel bolus, (f) silica gel bolus.

The head phantom fitted with thermoplastic underwent CT scans (GE Healthcare, Waukesha, WI, USA) with a slice thickness of 1.25 mm. The images were transferred to the pinnacle treatment planning system (Philips, Amsterdam, the Netherlands). We outlined the CTV nose, CTV ear and CTV parotid, separately. Then expand 0.5 cm margin to PTV which avoids exceeding the patient surface. In the plan optimization, PTV was used as objective structure and some ring around the PTV were made to constrain the dose outside PTV. Target volume of 95% is normalized to the prescription dose after optimization. These CT sets without a bolus were used to create the virtual bolus structure according to the PTV in the TPS [Fig. 1(a)], and the 3D Slicer version 4 software was used to extract bolus 3D point cloud data. Creo software (PTC, Boston, MA, USA) was used to reconstruct the 3D information to generate bolus images, which was converted and saved as a stereo lithography (STL) file, a commonly used file format in 3D printing. The data was further optimized and designed using Magics software (Materialise, Leuven, Belgium) [Fig. 1(b)], and the bolus shell and marker points were designed using Creosoftware and 3‐matic software (Materialise) [Fig. 1(c)]. Then, the final STL file of the bolus shell was printed with two copies in polylactic acid (PLA) by the MakerBot Replicator II printer (MakerBot Industries LLC, Brooklyn, NY, USA) [Fig. 1(d)], which took about 3–4 hr. The two shells were filled with hydrogel and silica gel separately. The hydrogel took about 10 min, but the silica gel needed about 10 hr at room temperature of 25°. The shells were removed by softening with a hot air gun after the solidification of hydrogel [Fig. 1(e)] and silica gel [Fig. 1(f)].

### 2.D Plan evaluation

The head phantom was kept in the same position and was fixed with thermoplastics in which the commercially bolus was placed outside the thermoplastic and the silica gel or hydrogel was placed inside the thermoplastic. Four sets of CT images were collected (thickness 2.5 mm): (a) without bolus, (b) commercial bolus, (c) silica gel bolus, and (d) hydrogel bolus. For the plan comparison, the PTV was outlined in the image of bolus‐free images and fused to the other three sets of CT images to ensure the consistency of the PTV. The IMRT plans which were given the same prescription dose (*V*_95%_ = 6000 cGy) were designed in CT images without a bolus and were copied to the other three plans. All the plans were re‐optimized under the condition that all parameters are consistent. In the plan optimization, collapsed cone convolution (CCC) algorithm was used for dose calculation and the objective value of maximum dose, uniform dose, minimum dose and minimum DVH were gave to the PTV. 0.3 × 0.3 cm dose calculation grid was choose in order to have sufficient sampling to determine depth of *D*_max_. We then performed an analysis and comparison of the four plans: the maximum size of the gap between skin and bolus, PTV *D*_max_, *D*_mean_, *D*_2%_, *D*_50%_, *D*_98%_, homogeneity index (HI, ((*D*_2% _− *D*_98%_)/*D*_50%_)), conformity index (CI, (TV_95% _× TV_95%_)/(*T *× *V*_95%_)),^5^ and comparison of the four Dose‐volume Histogram (DVH) graphs.

## 3 RESULTS

### 3.A Physical evaluation

**Figure 2:**
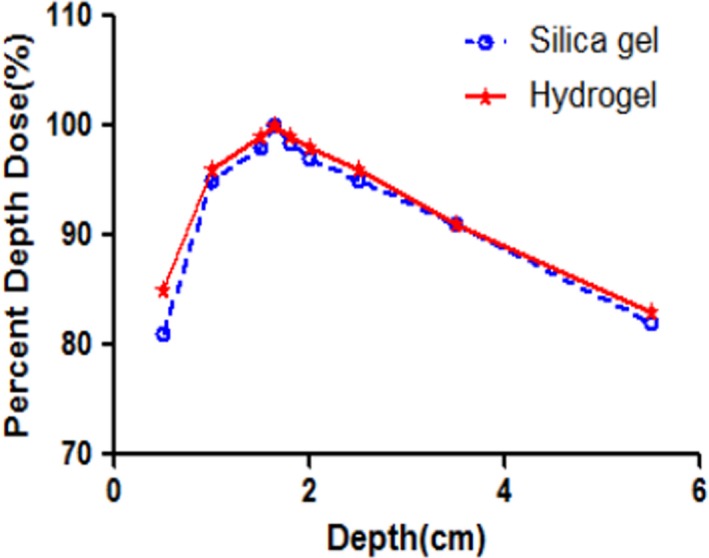
PDD curves at central axis comparing TPS‐calculated data for silica gel and hydrogel plans to the physical evaluation using the water‐equivalent phantom. The thicknesses of two boluses were 10 cm.

We compared the physical properties of two types of 3D printed bolus materials: silica gel and hydrogel. The mean density of silica gel and hydrogel were 1.15 and 1.04 g/cm^3^, respectively. The depth of the maximum dose (*d*_max_) for the silica gel and hydrogel boluses were 1.650 and 1.645 cm, respectively. There were good agreements between the PDD curves for the different fillings of 3D bolus materials (Fig. 2) with 10 cm thickness. The differences were less than 1.0% compared to the PDD data at all measurement points on the PDD curves, except D_0.5cm_ (the dose at a depth of 0.5 cm).

### 3.B Fabrication of boluses

**Figure 3:**
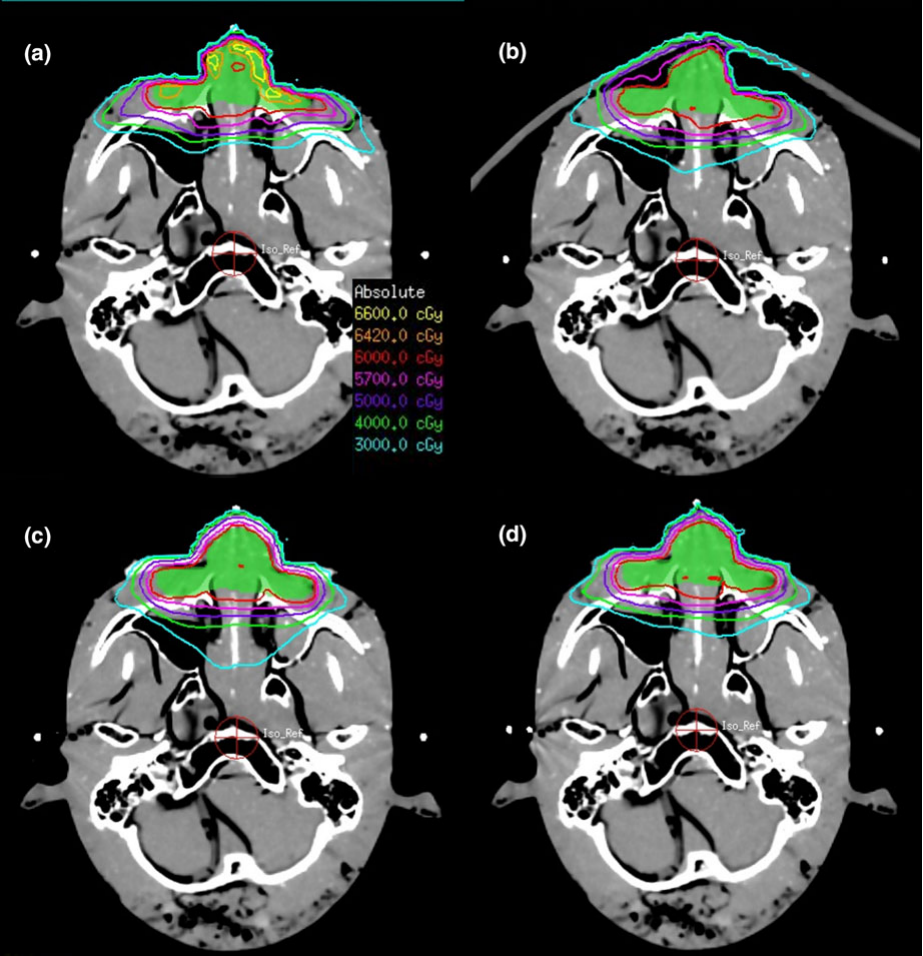
Comparison of plans for simulating nasal radiotherapy with different boluses. (a) Without bolus, (b) commercial bolus, (c) silica gel bolus, (d) hydrogel bolus

**Figure 4:**
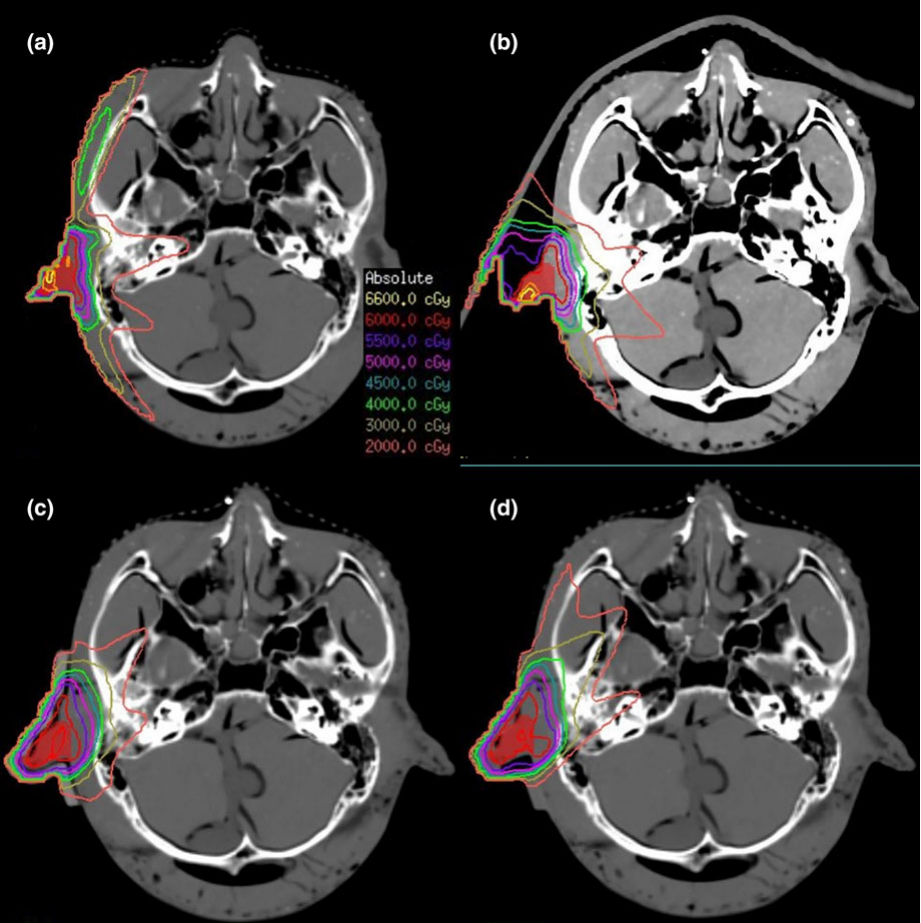
Comparison of plans for simulating ear radiotherapy with different materials. (a) Without bolus, (b) commercial bolus, (c) silica gel bolus, (d) hydrogel bolus.

We used a 3D reconstruction program to design the bolus shells, and the 3D printing technology was used to print the shells. The shells were filled with silica gel or hydrogel and then separated after curing to obtain the boluses. For the phantom treatment plans, the silicone gel, and hydrogel boluses were close to the mold body, and the outer U cover was fixed; thus the gap was small, and the maximum gap was 2 mm [Figs. 3(c), 3(d), 4(c), 4(d)]. The commercial bolus was a square‐shaped, large area, placed outside the positioning thermoplastic mask, which was not as well fixed in the location, less repeatable, and with a larger gap; the maximum gap was almost 1 cm [Figs. 3(b), 4(b)].

### 3.C Results of plan comparison

A head phantom was used to simulate nose, ear, and parotid tumor radiotherapy. In the absence of bolus, the dose near to the patient surface is insufficient, and the maximum dose will be increased in order to achieve a prescription dose of 95% of the target volume. In contrast, using of bolus pushes the build‐up region away from the patient, thus reducing the minimal dose to target and the 95% target volume is relatively easy to reach the prescribed dose so that the maximum dose is not required to increase (Table 1, Table 2). In nose treatment plans (Table 1, Fig. 3), the *D*_max_ (71.66 Gy) and HI (0.59) of PTV without a bolus were much higher than other plans, while the CI (0.63) was less than other plans, indicating that there were hot spots in the target and poor uniformity of target. When the boluses were used, the conditions were much better. We observed that the *V*_95%_ of silica gel bolus (99.86%) and hydrogel bolus (99.95%) were better than the commercial bolus (98.39%) and without a bolus (87.52%). Similarly, the HI and CI of the silica gel bolus (0.06; 0.79) and hydrogel bolus (0.058; 0.80) were better than the commercial bolus (0.094; 0.72) and without a bolus (0.59; 0.53). The bolus made using 3D printing skills had better results than the commercial boluses. The parameters of HI, CI, and *V*_95%_ of the hydrogel bolus plan were slightly better than that of the silica gel bolus.

**Table 1:** Parameters of nose radiotherapy

PTVnose	*D*_max_	*D*_mean_	*D*_2%_	*D*_50%_	*D*_98%_	HI	CI	*V*_95%_
Without bolus	7166	6002	6680	6116	3060	0.59	0.53	87.52
Commercial bolus	6419	6121	6328	6140	5750	0.094	0.72	98.39
Silica gel bolus	6456	6121	6318	6114	5950	0.060	0.79	99.86
Hydrogel bolus	6393	6106	6292	6100	5936	0.058	0.80	99.95

**Table 2:** Parameters of Ear radiotherapy

PTVear	*D*_max_	*D*_mean_	*D*_2%_	*D*_50%_	*D*_98%_	HI	CI	*V*_95%_
Without bolus	7312	6096	6950	6254	3650	0.53	0.51	82.51
Commercial bolus	6958	6135	6686	6176	4600	0.34	0.53	92.20
Silica gel bolus	6335	6086	6258	6082	5876	0.06	0.67	99.84
Hydrogel bolus	6103	6103	6306	6106	5800	0.08	0.56	99.10

**Figure 5:**
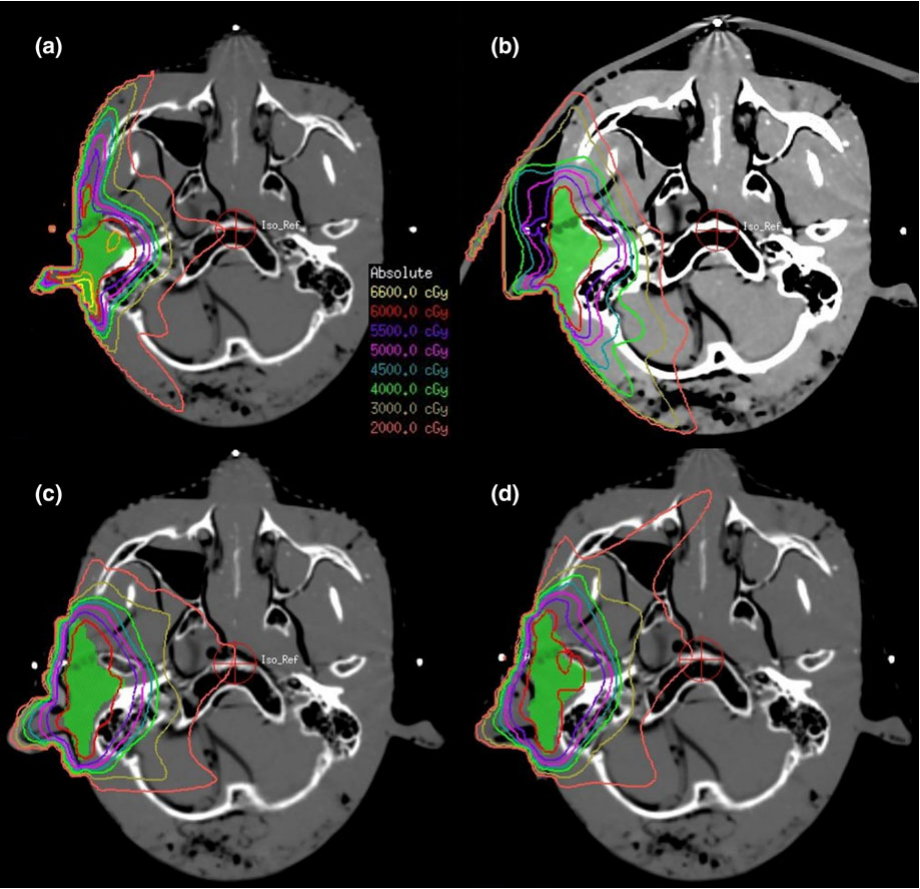
Comparison of plans for simulating parotid radiotherapy with different materials. (a): Without bolus, (b) commercial bolus, (c) silica gel bolus, (d) hydrogel bolus.

We could also conclude that the parameters of results (especially HI, CI, *V*_95%_) were better in 3D printing boluses than in commercially available boluses or without a bolus in ear and parotid plans. Two kinds of materials of 3D printing boluses were also compared in the simulated plans. In ear plans (Table 2, Fig. 4), the HI, CI, and *V*_95%_ of the hydrogel bolus plan were 0.08, 0.56, and 99.1%, respectively. Which were much better results than those of the commercial bolus (HI = 0.34, CI = 0.53, *V*_95% _= 92.20%), but only slightly superior to the silica gel bolus (HI = 0.06, CI = 0.67, *V*_95% _= 99.84%). In the parotid plan sets (Table 3, Fig. 5), the hydrogel bolus (HI = 0.07, CI = 0.69, *V*_95% _= 99.56%) and silica gel bolus (HI = 0.06, CI = 0.67, *V*_95% _= 99.65%) were slightly better than the commercial bolus (HI = 0.15, CI = 0.69, *V*_95% _= 96.12%). There was not much difference between the hydrogel bolus and silica gel bolus with respect to HI, CI, and *V*_95%_ values.

**Table 3:** Parameters of parotid radiotherapy

PTVparotid	*D*_max_	*D*_mean_	*D*_2%_	*D*_50%_	*D*_98%_	HI	CI	*V*_95%_
Without bolus	7022	6098	6670	6146	4700	0.32	0.55	88.79
Commercial bolus	6492	6109	6370	6162	5450	0.15	0.69	96.12
Silica gel bolus	6333	6092	6268	6080	5870	0.06	0.67	99.65
Hydrogel bolus	6331	6075	6268	6070	5848	0.07	0.69	99.56

*D*_max_ and *D*_mean_ represent the maximum and average values of target dose respectively; *D*_2%_, *D*_50%_ and *D*_98%_ represent the corresponding dose of target volume of 2%, 50%, and 98% respectively; HI = (*D*_2% _−* D*_98%_)/*D*_50%_.

### 3.D DVH curves

**Figure 6:**
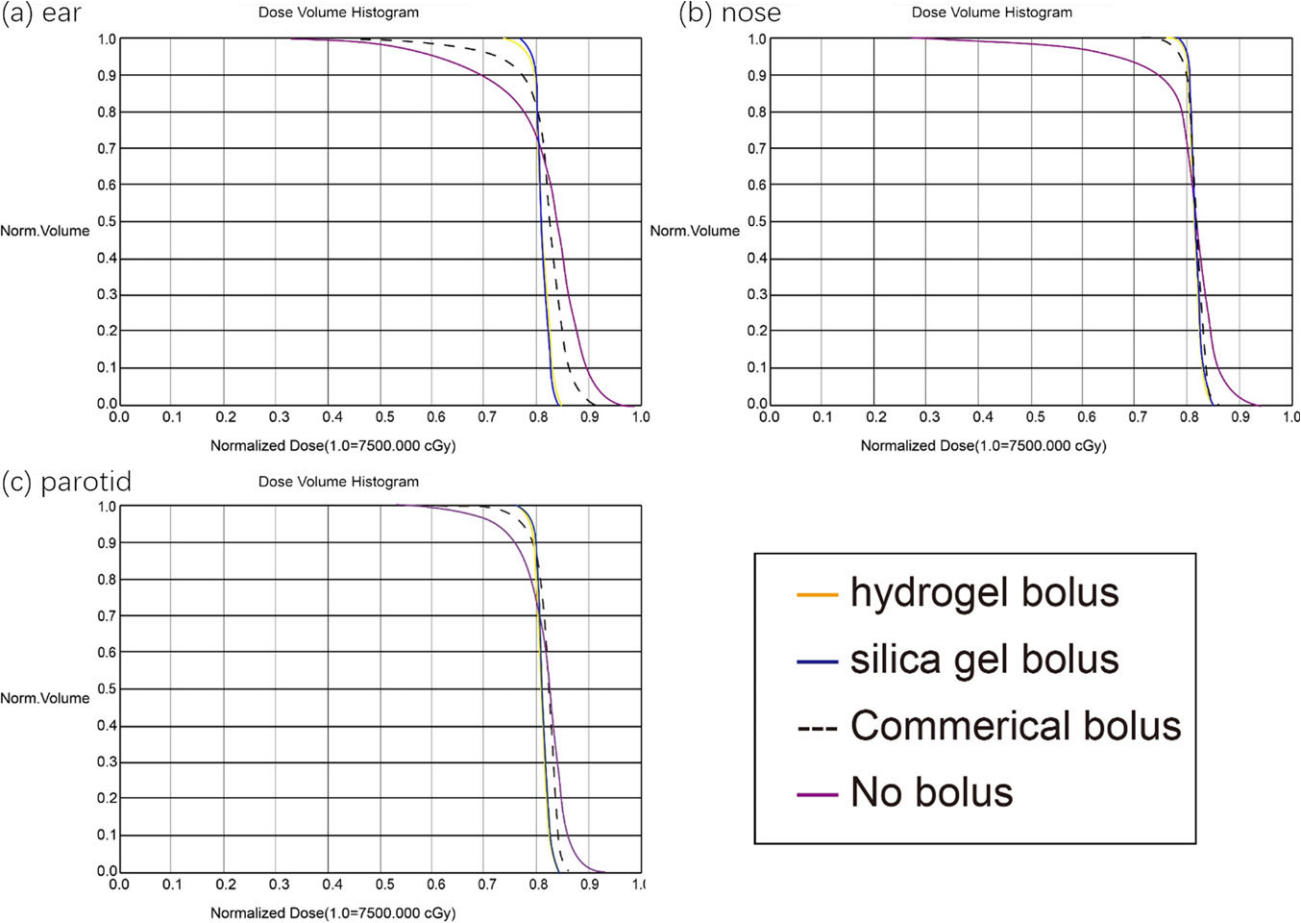
DVH curves for all the plans. (a) Ear plans, (b) nose plans, (c) parotid plans.

The DVH curves for all the plans (ear, nose, parotid) were shown in Fig. 6. In nose [Fig. 6(b)] and parotid plans [Fig. 6(c)], the DVH curves for the hydrogel bolus were slightly better than silica gel and both of them were significantly better than the commercial bolus. However, in the ear treatment plan, the DVH curves of silica gel were better than hydrogel, which was consistent with the parameters above.

## 4 DISCUSSION

In radiotherapy, the commercial boluses, which have different degrees of gaps because of their poor shape on the irregular surface. The effect of the air gap on surface dose reduction is related to factors such as field size, incident angle, ray energy, and patient characteristics.^4^ In recent years, with the continuous progress of 3D printing technology, 3D printing skills have becoming more and more widely used in the medical field, especially in plastic surgery, oral and maxillofacial surgery, and orthopedics.^6^ Application in the radiation therapy field has also gradually increased, especially in the production of boluses, most of which have been used in electron radiation therapy.^7^, ^8^, ^9^, ^10^ Indeed, many electron treatments do not involve a planning CT and instead rely entirely on a visual “clinical setup” of the patient anatomy within the room. This simplifies the procedure of making boluses, but we need to further facilitate accurate and reproducible alignment of patient anatomy.^11^ In this study, we used 3D printing skills to create individually customized boluses, which were designed to compensate for the irregular surface in photon IMRT radiotherapy.

In general, two ways have been reported of making a bolus in past studies. One method was to print a bolus directly with 3D printing materials after the design stage. Polylactic acid (PLA) whose physical density was 1.19 kg/m^3^, was a commonly used printing material, which had been demonstrated to be a bolus material in a previous study.^12^ Studies reported that the doses of 3D printed PLA bolus in phantom simulating radiotherapy of breast cancer after radical resection were more uniform than with the commercial bolus.^13^, ^14^ Acrylonitrile butadiene styrene (ABS) copolymer is another printing material commonly used except PLA, but both the two materials are too hard and with poor comfort. More importantly, the different infill percentage of these two materials corresponds to different densities, which may lead to discrepancies between the calculated and measured dose distribution.^15^ Another method is to print the shell of the bolus and then fill it with other soft materials. Richard et al.^16^ printed the shell in PLA using the 3D printer and filled it with silicone rubber for non‐melanoma skin cancer electron beam radiotherapy. Silicone rubber has the advantage when making a bolus due to its excellent biocompatibility, chemical stability, and good mechanical properties, but its density is 1.1–1.2 g/cm^3^ which differs from that of human tissue. In this study, we tried different filling materials, which were silica gel and hydrogel. Silica gel has the same physiochemical characteristics as silicone rubber. Hydrogels are widely used in biomedical fields, for example, scaffolds for tissue engineering, vehicles for drug delivery, actuators for optics and fluidics, and model extracellular matrices for biological studies.^17^ The density of hydrogel is similar to that of human tissue, but it is friable and of poor mechanical strength compared with silica gel or silicone rubber. Therefore, this study compares the dosimetric merits and demerits of two different materials.

In nose radiotherapy (Table 1), silica gel bolus and hydrogel bolus plans were much better than commercial bolus or without a bolus. The commercial bolus was a square with a thickness of 5 mm, which had a gap more than 6 mm when placed on an irregular surface [Figs. 3(b), 4(b)], but the air gaps of the 3D printed bolus were smaller, which was consistent with other studies.^3^ Although the boluses had the same conformation of nose, the dose distribution of the hydrogel bolus was more uniform and was slightly superior in *D*_max_, *D*_mean_, HI, CI, and *V*_95%_ than the silica gel bolus. We know the CI value is 0–1, and the greater the value, the better conformity; the HI value reflects the uniformity of dose in the target area, the lower the HI value, the better the homogeneity. We speculated that the advantages in the nose treatment plan in hydrogel maybe due to its similar density with human tissue. However, in the ear treatment, we observed that silica gel was better than hydrogel with respect to HI, CI, and *V*_95%_, and silica gel had smaller low‐dose areas (like 30 and 20 Gy dose areas) than hydrogel. It was possible that when the head model adopts a supine position, the hydrogel was more easily deformed and not closely jointed with the surface of the body because of gravity; behind the ear, the air gap was particularly large (Fig. 4). However, the hydrogel was much better than the commercially available bolus, because the commercial bolus and ear space exceed 1 cm with a poor contrast and a gravity effect (Fig. 4). In the parotid treatment, the plans for the two materials were quite similar and a bit better than the commercial one. The air gap between the commercial one and the surface was as big as in the ear treatment (Fig. 5).

To our knowledge, hydrogel is flexible, odorless, biologically nontoxic, and highly transparent, but it has not been used as a bolus in radiotherapy because of its physical characteristics. Hydrogels tend to lose water and undergo deformation, which is not suitable for long‐time use. The traditional polymer hydrogel is usually formed by chemical cross‐linking. The uneven dispersion of the chemical cross‐linking agent leads to an uneven gel network, and the gel is very fragile, which greatly limits its application.^18^, ^19^ We must solve these two problems for the clinical application of hydrogel. We used polyol polyurethane membrane to cover the hydrogel surface to prevent contact with air, thereby preventing dehydration. Because of its poor strength, many studies have reported methods to increase its strength, such as nanocomposite hydrogel^20^, ^21^ and double‐network hydrogel.^22^, ^23^, ^24^ Clinical application of strong, tough, and responsive hydrogels is the directions of future development, but more improvement is needed in biosafety and biocompatibility. Most of the novel hydrogels have strong hydrophilicity, which is not conducive to affinity with cells or biological tissues. Therefore, how to improve the biological function of hydrogel is also a problem to be overcome, or polymeric gel could be used which has been reported to have been used for bolus.^11^ In addition, the 3D printing materials which currently can be directly printed are relatively hard, and if we can directly print out 3D material which meets the special requirements of a bolus, we will greatly simplify the production process and promote commercialization. The next step is to try to use new materials to print tissue bolus, such as polycaprolactone (PCL),^25^ which has already been used in medical applications.

## 5 CONCLUSIONS

In this study, we used 3D printing skills to create individually customized boluses, which were designed to compensate for the irregular surface in photon IMRT radiotherapy. The dosimetric differences of hydrogel, silica gel, and commercial boluses were compared in head phantoms simulating nose, ear, and parotid gland radiotherapy. Silica gel and hydrogel boluses were not only good for fit and a high level of comfort and repeatability, but also had better dose parameters in IMRT plans. They may replace the commercial bolus for clinical use.

## CONFLICT OF INTEREST

The authors have no relevant conflict of interest to report.

## References

[1] Butson MJ , Cheung T , Yu P , et al. Effects on skin dose from unwanted air gaps under bolus in photon beam radiotherapy. Radiat Meas. 2000;32:201–204.

[2] Kong M , Holloway L . An investigation of central axis depth dose distribution perturbation due to an air gap between patient and bolus for electron beams. Australas Phys Eng Sci Med. 2007;30:111–119. 17682400 10.1007/BF03178415

[3] Fujimoto K , Shiinoki T , Yuasa Y , et al. Efficacy of patient‐specific bolus created using three‐dimensional printing technique in photon radiotherapy. Phys Med. 2017;38:1–9. 28610688 10.1016/j.ejmp.2017.04.023

[4] Vyas V , Palmer L , Mudge R , et al. On bolus for megavoltage photon and electron radiation therapy. Med Dosim. 2013;38:268–273. 23582702 10.1016/j.meddos.2013.02.007

[5] Barbiero S , Rink A , Matteucci F , et al. Single‐fraction flattening filter‐free volumetric modulated arc therapy for lung cancer: dosimetric results and comparison with flattened beams technique. Med Dosim. 2016;41:334–338. 27751617 10.1016/j.meddos.2016.09.002

[6] Tack P , Victor J , Gemmel P , et al. 3D‐printing techniques in a medical setting: a systemic literature review. BioMed Eng Online. 2016;15:115. 27769304 10.1186/s12938-016-0236-4 PMC5073919

[7] Kavanaugh JA , Hogstrom KR , Chu C , et al. Delivery confirmation of bolus electron conformal therapy combined with intensity modulated x‐ray therapy. Med Phys. 2013;40:021724. 23387747 10.1118/1.4788657

[8] Carver RL , Hogstrom KR , Chu C , et al. Accuracy of pencil‐beam redefinition algorithm dose calculations in patient‐like cylindrical phantoms for bolus electron conformal therapy. Med Phys. 2013;40:071720. 23822424 10.1118/1.4811104

[9] Su S , Moran K , Robar JL , et al. Design and production of 3D printed bolus for electron radiation therapy. J Appl Clin Med Phys. 2014;15:4831. 25207410 10.1120/jacmp.v15i4.4831 PMC5875499

[10] Zou W , Fisher T , Zhang M , et al. Potential of 3D printing technologies for fabrication of electron bolus and proton compensators. J Appl Clin Med Phys. 2015;16:4959. 26103473 10.1120/jacmp.v16i3.4959 PMC5690113

[11] Adamson JD , Cooney T , Demehri F , et al. Characterization of water‐clear polymeric gels for use as radiotherapy bolus. Technol Cancer Res Treat. 2017;16:923–929. 10.1177/1533034617710579 PMC5762050 28554255

[12] Burleson S , Baker J , Hsia AT , et al. Use of 3D printers to create a patient‐specific 3D bolus for external beam therapy. J Appl Clin Med Phys. 2015;16:166–178. 10.1120/jacmp.v16i3.5247 PMC5690114 26103485

[13] Ha JS , Jung JH , Kim MJ , et al. Customized 3D printed bolus for breast reconstruction for modified radical mastectomy (MRM). Prog Med Phys. 2016;27:196–202.

[14] Park SY , Choi CH , Park JM , et al. A patient‐specific polylactic acid bolus made by a 3d printer for breast cancer radiationtherapy. PLoS ONE. 2016;11:0168063. 10.1371/journal.pone.0168063 PMC5145239 27930717

[15] Ricotti R , Ciardo D , Pansini F , et al. Dosimetric characterization of 3D printed bolus at different infill percentage for external photon beam radiotherapy. Phys Med. 2017;39:25–32. 28711185 10.1016/j.ejmp.2017.06.004

[16] Canters RA , Lip IM , Wendling M , et al. Clinical implementation of 3D printing in the construction of patient specific bolus for electron beam radiotherapy for non‐melanoma skin cancer. Radiother Oncol. 2016;121:148–153. 27475278 10.1016/j.radonc.2016.07.011

[17] Sun JY , Zhao X , Illeperuma WR , et al. Highly stretchable and tough hydrogels. Nature. 2012;489:133–136. 22955625 10.1038/nature11409 PMC3642868

[18] Okay O , Oppermann W . Polyacrylamide‐clay nanocomposite hydrogels: rheological and light scattering characterization. Macromolecules. 2007;40:3378–3387.

[19] Shibayama M , Karino T , Miyazaki S , et al. Small‐angle neutron scattering study on uniaxially stretched poly (N‐isopropylacrylamide)‐clay nanocomposite gels. Macromolecules. 2005;38:10772–10781.

[20] Yang J , Han CR , Duan JF , et al. Synthesis and characterization of mechaniacally flexible and tough cellulose nanocrystals‐polyacrylamide nanocomposite hydrogels. Cellulose. 2013;20:227–237.

[21] Yang J , Han CR , Xu F , et al. Simple approach to reinforce hydrogels with cellulose nanocrystals. Nanoscale. 2014;6:5934–5943. 24763379 10.1039/c4nr01214c

[22] Hu Z , Chen G . Novel nanocomposite hydrogels consisting of layered double hydroxide with ultrahigh tensibility and hierarchical porous structure at low inorganic conten. Adv Mater. 2014;26:5950–5956. 24923256 10.1002/adma.201400179

[23] Gao G , Du G , Cheng Y , et al. Tough nanocomposite double network hydrogels reinforced with clay nanorods through covalent bonding and reversible chain adsorption. J Mater Chem B. 2014;2:1539–1548. 10.1039/c3tb21554g 32261372

[24] Du G , Gao G , Hou R , et al. Tough and fatigue resistant biomi‐metic hydrogels of interlaced self‐assembled conjugated polymerbelts with a polyelectrolyte network. Chem Mater. 2014;26:3522–3529.

[25] Morrison RJ , Hollister SJ , Niedner MF , et al. Mitigation of trachebronchomalacia with 3D‐printed personalized medical devices in pediatric patients. Sci Transl Med. 2015;7:285–264. 10.1126/scitranslmed.3010825 PMC4495899 25925683

